# The Association Between Lifestyle Factors and Sleep Quality in Haemodialysis Patients

**DOI:** 10.1002/nop2.70768

**Published:** 2026-08-21

**Authors:** Lu Zhang, Sumei Zhang, Xuanbing Tang

**Affiliations:** ^1^ School of Nursing and Rehabilitation Xi'an Medical University Xi'an Shaanxi People's Republic of China

**Keywords:** haemodialysis, lifestyle, sleep quality

## Abstract

**Aims:**

To evaluate sleep quality in patients receiving maintenance haemodialysis and to identify the association between lifestyle factors and sleep quality.

**Design:**

A cross‐sectional study design was employed.

**Methods:**

In this study, 286 haemodialysis patients from two hospitals were selected. Logistic regression analysis was used to identify the association between lifestyle factors and sleep quality.

**Results:**

The mean Pittsburgh Sleep Quality Index was (7.89 ± 4.68). 73.4% of patients had poor sleep quality. In multivariate logistic analyses, age, maintenance haemodialysis duration, depression (psychological factor) and self‐management (as a behavioural lifestyle domain) were major factors associated with sleep quality.

**Patient or Public Contribution:**

No patients or members of the public were involved in the design, conduct, interpretation, or dissemination of this study. Participants were involved solely as respondents in the data collection process.

**Implications for Nursing Practice:**

This study found that depression and self‐management were the key factors associated with sleep quality in patients undergoing maintenance haemodialysis. Based on these findings, we recommend the following nursing practices: First, nurses should integrate brief sleep‐quality screening and depression screening into routine dialysis care to facilitate early identification of at‐risk patients. Second, nursing interventions should strengthen patients' self‐management skills, including dietary adherence, fluid restriction and medication compliance, as these may contribute to better sleep outcomes. Third, dialysis teams should adopt a holistic approach that addresses both psychological well‐being and behavioural self‐management, recognizing their combined influence on patients' sleep quality. Fourth, nurses should provide individualized lifestyle counselling tailored to each patient's dietary and fluid needs, recognizing that active participation in treatment plans may improve sleep quality.

## 1 Introduction

Lifestyle is one of the most important factors affecting health. A healthy lifestyle can promote health; on the contrary, an unhealthy lifestyle can lead to the occurrence of lifestyle‐related diseases, such as hypertension, diabetes and dyslipidemia, which can contribute to the development of chronic kidney disease, thus increasing the number of patients undergoing maintenance haemodialysis (MHD) (Hara et al. 2021).

In relevant studies of other populations, lifestyle factors have been linked to sleep disturbance. For example, the study by Gangwisch et al. suggested that high glycemic index and high sugar diet induce compensatory hyperinsulinemia, thus inducing reflex secretion of adrenaline, cortisol, glucagon, growth hormone and other counter‐regulatory hormones, which easily leads to insomnia (Gangwisch et al. 2020). In addition, a survey on the sleep quality of adolescents has shown that high plasma docosahexaenoic acid (DHA) is associated with earlier bedtime and longer weekend sleep (Jansen et al. 2020). Long‐term physical activity has a positive effect on sleep, and as exercise duration and step count increase, sleep quality can be improved. Even activities with minimal physical activity can have a positive effect on sleep (Sullivan Bisson et al. 2019). Regarding the relationship between alcohol, smoking and sleep quality, research has shown that sleep disorders are more common among alcohol abusers (Zhang et al. 2021). In addition, any form of nicotine intake, such as patches, medication and smoking, can lead to sleep disorders, reducing total sleep time and prolonging sleep onset latency (AlRyalat et al. 2021).

Haemodialysis is the primary treatment modality for prolonging survival of patients with end‐stage renal disease (ESRD) (Johansen et al. 2021; Verberne et al. 2016). For example, in the United States, nearly 90% of ESRD patients receive haemodialysis treatment based on dialysis centres (Johansen et al. 2021). In long‐term dialysis treatment, maintaining a healthy lifestyle is an important responsibility for patients and healthcare providers, and also an important measure to improve patient prognosis (Su et al. 2022). Healthy lifestyles in patients with mild to moderate chronic kidney disease are associated with lower all‐cause mortality and cardiovascular disease risk (Schrauben et al. 2021). A 4‐year prospective cohort study on MHD patients shows that a healthy lifestyle can reduce the all‐cause mortality and cardiovascular mortality of patients by 36% and 35% respectively (Su et al. 2022).

For patients on maintenance haemodialysis, the incidence of sleep disorders is high (Kalantar‐Zadeh et al. 2022; Scherer et al. 2017), mainly manifested as insomnia, fragmented sleep, frequent nocturnal awakenings, periodic limb movements, apnea syndrome, etc. (Scherer et al. 2017). Sleep disorders, which can seriously affect the quality of life and prognosis of MHD patients, can increase the risk of all‐cause mortality (Han et al. 2021). However, to our knowledge, there have been few studies on the association between lifestyle and sleep quality on MHD patients.

Broadly, the definition of lifestyle factors includes physical activity level, smoking status, alcohol intake, nutritional status, intake of healthy/unhealthy foods, sexual health and environmental health (Zaman et al. 2019). However, the concept of “lifestyle” is inherently complex and lacks a universally accepted consensus definition across scientific disciplines, having been used and defined in different ways in various fields of knowledge (Brivio et al. 2023). According to the World Health Organization, lifestyle is a way of living which is based on identifiable patterns of behaviour, and these patterns are determined by the interplay between individual, social and environmental factors (Ding et al. 2016; Trujillo‐Cáceres et al. 2026). From a sociological perspective, healthy lifestyles are defined as collective patterns of health‐related behaviour based on choices from options available to people according to their life chances (Cockerham 2005). In epidemiological and behavioural medicine research, lifestyle is typically operationalized through measurable behavioural parameters, including physical activity, dietary intake, alcohol consumption and smoking (Kvaavik et al. 2010). A systematic taxonomy of health‐related behaviours further identifies six main categories relevant to longevity: diet/exercise, vitality/stress, sleep, cognition, substance use and other risk (Salvador‐Carulla et al. 2013). In addition, psychological distress such as depression has been recognized as an important indirect factor closely linked to health‐related lifestyle (Blair et al. 2019). Based on the above conceptual framework, the lifestyle factors focused on in this study were defined as nutritional status, physical activity, disease self‐management, smoking and drinking history. Depression was regarded as a critical psychological influencing factor included in the analysis.

Although previous studies have examined lifestyle factors and sleep quality in haemodialysis populations, most have focused on individual lifestyle behaviours in isolation, such as physical activity or nutritional status, without considering the interplay between psychological status and disease‐specific self‐management. Furthermore, few studies have simultaneously adjusted for multiple lifestyle domains and psychological covariates in a single multivariable model. The present study adds to the existing literature by: (1) incorporating both behavioural lifestyle domains (nutritional status, physical activity, self‐management, smoking and drinking) and psychological status (depression) within a unified analytical framework; (2) utilizing validated instruments including the Pittsburgh Sleep Quality Index (PSQI), Malnutrition‐Inflammation Score (MIS), Haemodialysis Self‐Management Instrument (HDSMI), and Beck Depression Inventory‐II (BDI‐II); and (3) applying multivariate logistic regression to identify independent factors associated with sleep quality after adjusting for potential confounders. This integrated approach may provide a more comprehensive understanding of the factors influencing sleep quality in patients undergoing maintenance haemodialysis.

## 2 The Study

### 2.1 Design

A cross‐sectional study design was employed. In our study, 286 patients receiving maintenance haemodialysis from two hospitals were investigated. We investigated the sleep quality of patients through the Pittsburgh Sleep Quality Index (PSQI) and understood their lifestyle status by investigating nutrition, depression, self‐management, exercise, smoking history and drinking history.

### 2.2 Inclusion and Exclusion Criteria

Participants were eligible for this study if they met the following criteria: (1) having been receiving maintenance haemodialysis (MHD) for at least 3 months; (2) being 18 years of age or above. Individuals were excluded from the study in the presence of: (1) any acute medical or surgical illnesses requiring hospital admission; (2) a diagnosis of dementia or any psychotic disorder.

### 2.3 Instrument

Data for this study were collected via self‐administered questionnaires. The questionnaires included items assessing sociodemographic characteristics, sleep quality and multiple lifestyle domains, such as nutritional status, depression, exercise status, self‐management, smoking history and drinking history.

#### 2.3.1 Sociodemographic and Clinical Characteristics

Sociodemographic and clinical characteristics comprised age, gender, education level, marital status, employment status, duration of maintenance haemodialysis (MHD), primary renal diagnosis, body mass index (BMI), systolic blood pressure (SBP) and diastolic blood pressure (DBP). BMI was computed as weight in kilograms divided by height in metres squared (kg/m^2^). Blood pressure was assessed prior to the dialysis session. Laboratory data including haemoglobin (Hb), calcium (Ca) and phosphate (P) were obtained at study enrollment.

#### 2.3.2 Evaluation of Sleep Quality

Sleep quality was evaluated using the Pittsburgh Sleep Quality Index (PSQI). This scale is composed of 19 self‐rated items that evaluate 7 subscales: subjective sleep quality, sleep latency, sleep duration, habitual sleep efficiency, sleep disturbances, use of sleep medication and daytime dysfunction. Participants were instructed to report on their sleep patterns over the preceding month. The total PSQI score ranges from 0 to 21, with higher scores reflecting worse sleep. A score of > 5 is typically interpreted as indicative of poor sleep quality, while a score ≤ 5 signifies good sleep (Buysse et al. 1989). In the present sample, the Cronbach's alpha for the PSQI was 0.822.

#### 2.3.3 Assessment of Lifestyle Factors

(1) Smoking history and drinking history.

In this study, these two items assessed whether patients had a history of smoking and drinking, without investigating their specific smoking and alcohol consumption. Smoking and drinking history were assessed using binary (yes/no) questions. We opted for this simplified assessment given that detailed quantity‐frequency measures are subject to considerable recall bias in this patient population, and our primary aim was to capture the presence of these behaviours as lifestyle indicators.

(2) Exercise status.

In this study, this item only investigated whether patients engaged in regular physical exercise, without covering specific exercise duration or forms.

(3) Evaluation of nutrition.

The Malnutrition‐Inflammation Score (MIS) was employed to assess patients' nutritional status. This tool includes four major sections: medical history, physical examination, BMI and laboratory investigations. Weight change, dietary intake, gastrointestinal symptoms, functional status, dialysis vintage and complications were recorded in the medical history. Physical examination focused on subcutaneous fat depletion and muscle atrophy. Serum albumin and total iron‐binding capacity (or transferrin) served as laboratory indicators. Each of the 10 items is rated on a 4‐point scale (0–3), and a higher total score reflects more severe malnutrition (Kalantar‐Zadeh et al. 2001). In the present sample, the Cronbach's alpha for the MIS was 0.671.

During the assessment, data on dietary intake, gastrointestinal symptoms and functional capacity were recorded according to patients' self‐reported conditions. Weight change was documented based on objective weight measurements. Serum albumin and total iron‐binding capacity (or transferrin) were obtained from recent laboratory results within the previous 3–6 months. Subcutaneous fat loss was evaluated by measuring triceps skinfold thickness (TSF) using a skinfold calliper (Sedhain et al. 2015). Mid‐upper arm circumference (MAC) serves as a reliable indicator of fat‐free muscle mass depletion. MAC was measured using a non‐stretchable tape at the midpoint between the acromion of the scapula and the olecranon of the ulna (Sedhain et al. 2015).

(4) Evaluation of self‐management.

The Haemodialysis Self‐Management Instrument (HDSMI) was adopted to assess patients' self‐management ability. This instrument comprises four components: problem‐solving ability, emotional regulation, self‐care practice and collaborative partnership. It contains 20 items; each scored on a 4‐point Likert scale ranging from 1 (never) to 4 (always). The total score ranges from 20 to 80, and higher overall scores indicate a higher level of self‐management (Li et al. 2014). In the present sample, the Cronbach's alpha for the HDSMI was 0.919.

(5) Evaluation of depression.

The Beck Depression Inventory‐II (BDI‐II) includes 21 items, each scored from 0 to 3 points. A higher total score indicates more severe depression. Those with a total score of 13 or below are classified as mild depression, those with a score of 14–20 are classified as moderate depression, and those with a score of 21 or above are classified as severe depression (Beck et al. 1996). In the present sample, the Cronbach's alpha for the BDI‐II was 0.926.

### 2.4 Data Collection

Data collection was performed via face‐to‐face interviews, anthropometric measurements and reviews of electronic medical records.

### 2.5 Statistical Analyses

Continuous variables were presented as the mean ± standard deviation (SD), and categorical variables were summarized as frequencies and percentages. Group comparisons for continuous data were performed using independent *t*‐tests or Mann–Whitney U tests according to data distribution. The *χ* ^2^ test was applied for comparisons of categorical variables. Variables with significant differences in univariate analysis were entered into the multivariate logistic regression model. Multivariate logistic regression was conducted to adjust for potential confounders and explore independent lifestyle factors associated with sleep quality. Sleep quality was entered as the dependent variable, coded as 1 = good sleep quality (PSQI ≤ 5) and 2 = poor sleep quality (PSQI > 5), with good sleep quality serving as the reference category. OR > 1 indicates higher odds of poor sleep quality. Smoking and drinking were retained in the model due to clinical relevance. Results were reported using odds ratios (OR) with 95% confidence intervals (CI). Multicollinearity among independent variables was assessed using variance inflation factors (VIF) derived from linear regression, with VIF > 10 indicating potential collinearity. Model calibration was evaluated using the Hosmer‐Lemeshow goodness‐of‐fit test, with *p* > 0.05 indicating acceptable model fit. Model discrimination was assessed using the area under the receiver operating characteristic curve (AUC). A *p*‐value < 0.05 was considered statistically significant, and all statistical analyses were performed using SPSS version 16.0 (SPSS Inc., Chicago, IL, USA).

### 2.6 Ethical Consideration

This study involved human participants and was approved by the Medical Ethics Review Committee of Xi'an Medical University (No. XYLS2024220). All procedures performed in studies involving human participants were in accordance with the ethical standards of the institutional research committee and with the 1964 Helsinki Declaration and its later amendments.

Written informed consent was obtained from all participants prior to enrollment. Participation was entirely voluntary, and all personally identifiable information was kept confidential to protect participants' privacy.

## 3 Results

We studied 286 MHD patients aged (57.51 ± 16.05) years, of whom 66.8% (*n* = 191) were men. More than half (65.0%) of the patients had a low education level. The primary disease of ESRD was diabetic nephropathy (*n* = 113, 39.5%). Other causes were hypertensive nephropathy (*n* = 67, 23.4%), glomerulonephritis (*n* = 58, 20.3%) and other kidney diseases (*n* = 48, 16.8%). The characteristics are shown in Table 1.

**TABLE 1:** Comparison of general information between the two groups.

Characteristic	Total	Good sleepers (PSQI ≤ 5) (*n* = 76)	Poor sleepers (PSQI > 5) (*n* = 210)	*t*/*Z*/$\chi^{2}$	*p*
Age, years	57.51 ± 16.05	52.70 ± 15.90	59.28 ± 15.78	−3.102	0.002
Gender, *n* (%)				0.850	0.356
Male	191 (66.8)	54 (71.1)	137 (65.2)		
female	95 (33.2)	22 (28.9)	73 (34.8)		
Education, *n* (%)				7.003	0.008
High school and below	186 (65.0)	40 (52.6)	146 (69.5)		
College and above	100 (35.0)	36 (47.4)	64 (30.5)		
Marriage, *n* (%)				0.252	0.615
Married	189 (66.1)	52 (68.4)	137 (65.2)		
Others(single, divorce, widowed)	97 (33.9)	24 (31.6)	73 (34.8)		
Employment, *n* (%)				5.754	0.056
Working	53 (18.5)	21 (27.6)	32 (15.2)		
Retired	102 (35.7)	25 (32.9)	77 (36.7)		
Others (unemployed、students)	131 (45.8)	30 (39.5)	101 (48.1)		
MHD duration, months	35.44 ± 30.07	45.21 ± 34.98	31.88 ± 27.22	3.006	0.003
Primary renal diagnosis, *n* (%)				9.043	0.029
Glomerulonephritis	58 (20.3)	22 (28.9)	36 (17.1)		
Diabetic nephropathy	113 (39.5)	20 (26.3)	93 (44.3)		
Hypertensive nephropathy	67 (23.4)	19 (25.0)	48 (22.9)		
Other*	48 (16.8)	15 (19.7)	33 (15.7)		
BMI	21.82 ± 3.38	21.89 ± 2.69	21.80 ± 3.60	0.194	0.846
SBP(mmHg)	150.92 ± 18.76	149.22 ± 17.60	151.56 ± 19.18	−0.917	0.360
DBP(mmHg)	88.41 ± 51.32	85.77 ± 12.87	89.38 ± 59.54	−0.513	0.608
Ca (mmol/L)	1.99 ± 0.45	2.04 ± 0.47	1.97 ± 0.44	−1.420	0.156
P (mmol/L)	1.82 ± 0.58	1.77 ± 0.51	1.84 ± 0.61	−0.807	0.421
Hb (g/L)	100.28 ± 22.83	100.54 ± 23.15	100.19 ± 22.79	0.101	0.920

*Note:* * obstructive nephropathy, polycystic kidney, lupus nephritis, nephritic syndrome, infective endocarditis, interstitial nephritis, IgA nephropathy, stenosis of renal artery, renal tuberculosis, gouty nephropathy.

Abbreviations: BMI:Body mass index; Ca: calcium; DBP: diastolic blood pressure; Hb: haemoglobin: MHD: maintenance haemodialysis; P:phosphate; PSQI: Pittsburgh Sleep Quality Index; SBP:systolic blood pressure.

### 3.1 Comparison of General Characteristics Between the Two Groups

Regarding baseline characteristics, no significant between‐group differences were observed in terms of gender, marital status, employment status, BMI, blood pressure, calcium, phosphate, or haemoglobin levels. Patients with poor sleep quality were older (*p* = 0.002), had a lower educational level (*p* = 0.008), and a shorter duration of maintenance haemodialysis (*p* = 0.003) than those with good sleep quality. In addition, diabetic nephropathy as the primary renal diagnosis was more prevalent in the poor sleep group (*p* = 0.029). (Table 1).

### 3.2 Comparison of Lifestyle Differences Between the Two Groups

The mean PSQI score was (7.89 ± 4.68). 210 (73.4%) patients had poor sleep quality. The mean MIS, BDI and self‐management score were (8.47 ± 2.77), (14.67 ± 10.09) and (55.36 ± 11.13), respectively.

Regarding lifestyle‐related variables, there were no significant between‐group differences in smoking or drinking history. Patients with poor sleep quality demonstrated significantly lower self‐management scores (*p* = 0.002) and were less likely to engage in regular exercise (*p* = 0.006) compared with those with good sleep quality. In addition, the poor sleep group had significantly higher MIS (*p* = 0.005) and BDI score (*p* < 0.001) (Table 2).

**TABLE 2:** Comparison of lifestyle differences between the two groups.

Characteristic	Total	Good sleepers (PSQI ≤ 5) (*n* = 76)	Poor sleepers (PSQI > 5) (*n* = 210)	*t*/*Z*/$\chi^{2}$	*p*
MIS	8.47 ± 2.77	7.71 ± 2.38	8.75 ± 2.85	−2.835	0.005
BDI scores	14.67 ± 10.09	10.00 ± 9.12	16.36 ± 9.90	−5.255	< 0.001
Self‐management score	55.36 ± 11.13	58.93 ± 11.81	54.07 ± 10.61	−3.051	0.002
Exercise status, *n* (%)				7.540	0.006
Exercise	173 (60.5)	56 (73.7%)	117 (55.7%)		
No exercise	113 (39.5)	20 (26.3%)	93 (44.3%)		
Smoking history, *n* (%)				0.368	0.544
Yes	83 (29.0)	20 (26.3)	63 (30.0%)		
No	203 (71.0)	56 (73.7)	147 (70.0%)		
Drinking history, *n* (%)				1.312	0.252
Yes	49 (17.1)	16 (21.1)	33 (15.7)		
No	237 (82.9)	60 (78.9)	177 (84.3)		

Abbreviations: BDI: Beck Depression Inventory; MIS: Malnutrition‐Inflammation Score; PSQI: Pittsburgh Sleep Quality Index.

### 3.3 Multivariate Logistic Regression

Multivariate logistic regression analysis was performed with sleep quality (good vs. poor) as the dependent variable. Independent variables included those showing significant differences in univariate analysis: age, educational level, duration of MHD, primary renal diagnosis, MIS, BDI, self‐management score and physical activity. Smoking and drinking history were also incorporated as lifestyle covariates.

Multivariate logistic regression revealed that age (*p* = 0.026), MHD duration (*p* = 0.003), BDI score (*p* < 0.001) and self‐management score (*p* = 0.030) were independently associated with sleep quality. Age and BDI score were associated with higher odds of poor sleep quality (OR > 1). In contrast, longer MHD duration and higher self‐management score were associated with lower odds of poor sleep quality (OR < 1). Educational level, primary renal diagnosis, nutritional status (MIS), exercise status, smoking history and drinking history showed no independent associations in the adjusted model (Table 3).

**TABLE 3:** Multivariate factors logistic regression on sleep quality.

	B	SE	Wald $\chi^{2}$	*p*	OR	95% CI
Age	0.022	0.010	4.951	0.026	1.023	1.003 ~ 1.043
Education	−0.545	0.327	2.774	0.096	0.580	0.305 ~ 1.101
MHD duration	−0.015	0.005	8.763	0.003	0.985	0.975 ~ 0.995
Primary renal diagnosis	0.122	0.148	0.682	0.409	1.130	0.845 ~ 1.511
MIS	0.060	0.063	0.925	0.336	1.062	0.939 ~ 1.201
BDI	0.079	0.021	14.084	< 0.001	1.082	1.038 ~ 1.127
Self‐management score	−0.032	0.015	4.704	0.030	0.969	0.941 ~ 0.997
Exercise status	0.351	0.342	1.051	0.305	1.420	0.726 ~ 2.776
Smoking history	−0.507	0.422	1.447	0.229	0.602	0.264 ~ 1.376
Drinking history	0.758	0.474	2.562	0.109	2.135	0.843 ~ 5.404
Constant	0.223	1.423	0.025	0.876	1.250	

Abbreviations: 95% CI: 95% confidence interval; B: regression coefficient; BDI: Beck Depression Inventory; MHD: maintenance haemodialysis; MIS: Malnutrition‐Inflammation Score; OR: Odds Ratio; SE: standard error.

The VIF values for all independent variables ranged from 1.012 to 1.372, indicating no significant multicollinearity. The Hosmer‐Lemeshow test yielded a χ^2^ value of 4.162 (*p* = 0.842), indicating acceptable model fit. The AUC was 0.794 (*p* < 0.001), suggesting good discriminatory ability of the model.

After adjustment, BDI (as a psychological factor) and self‐management (as a behavioural domain) remained significant lifestyle‐related factors. These findings indicate that psychological status and self‐management capacity are key factors associated with sleep quality among maintenance haemodialysis patients.

## 4 Discussion

Maintaining a healthy lifestyle is the core of managing kidney disease patients, and unhealthy lifestyle behaviours are modifiable, which can significantly impact disease progression and improve prognosis. Similarly, lifestyle is related to the patient's sleep quality (Schrauben et al. 2022).

Our study showed that 210 (73.4%) patients had poor sleep quality. According to a research report, the incidence of sleep disorders in dialysis patients is as high as 86% (Khamis et al. 2020). Another study of dialysis patients in China found that the incidence of sleep disorders was 61.5% (Han et al. 2021). Various factors contribute to the high incidence of sleep disorders in haemodialysis patients, including interdialytic weight gain, dialysis modality, cholesterol and haemoglobin levels, parathyroid hormone levels, medication use and impaired respiratory function.

In our study, the relationship between depression and sleep quality was significant in both univariate and multivariate analyses (Tables 2 and 3). Haemodialysis significantly impacts patients' lives, leading to reduced functional capacity, social isolation, loss of self‐confidence and ultimately, hopelessness about the future. In addition to experiencing physiological changes, patients also face psychological pressure, both of which can affect patients' mental state and sleep quality (Dashtidehkordi et al. 2019). Therefore, most patients cannot adapt to physiological and lifestyle changes, resulting in anxiety, depression and isolation (Dashtidehkordi et al. 2019), which affect their sleep quality. Evidence suggests that comprehensive dialysis care, psychological counselling and structured health education may help alleviate depressive symptoms and are associated with better sleep quality among haemodialysis patients (Al Naamani et al. 2021).

In this study, self‐management is also an important lifestyle factor associated with sleep quality (Tables 2 and 3). Dialysis patients must effectively manage multiple aspects of their care, including fluid intake, diet, medication adherence, dialysis scheduling and balancing daily life with treatment demands. Good health education and a proper understanding of dialysis treatment can enhance patients' treatment adherence, thereby improving their clinical outcomes and quality of life, and ultimately enhancing their prognosis (Ma et al. 2022). Actively conducting home‐based self‐observation and disease self‐management has become a key measure to improve the quality of life and prognosis of dialysis patients. A study investigated the impact of a self‐management program based on the 5A nursing model—which provides nursing intervention through assessment, advice, agreement, assistance and arrangement stages—on the quality of life of patients undergoing haemodialysis. The results showed that after 3 months of implementing the 5A nursing model, the sleep quality dimension score on the quality of life scale was significantly higher in the intervention group than in the control group, and the difference was statistically significant (Keivan et al. 2023). Each patient is the primary manager of their own disease and treatment, and must apply the nursing knowledge learned from medical staff to active self‐management (Stevenson et al. 2018). Higher levels of self‐management enable patients to become active participants in their treatment plans, allowing them to better align their care with their metabolic needs and personal lifestyle preferences, which may in turn improve sleep quality.

This study still has some limitations. First, this study is a cross‐sectional study and cannot confirm the causal relationship between lifestyle and sleep quality. Second, because the patients with psychotic disorder and dementia were excluded from the study, the present results cannot be applied to these populations. In addition, most of our data come from patients' self‐reports, which may have memory bias. Third, we have only provided framework recommendations for lifestyle, and the specific implementation details still need further research. Fourth, smoking, alcohol use and physical activity were assessed using brief yes/no questions to reduce participant burden and to minimize recall bias. However, we acknowledge that this approach did not capture detailed information on frequency, duration, intensity, or quantity, which may limit measurement precision. Future studies using validated, detailed instruments are warranted to further explore these relationships.

## 5 Conclusions

Our findings suggest that depression and self‐management are most strongly associated with sleep quality. Therefore, healthcare providers in dialysis units should fully recognize the close links between psychological status, self‐management ability and sleep among patients undergoing maintenance haemodialysis.

## Author Contributions

**Lu Zhang:** conceptualization, methodology, software, data curation, investigation, validation, formal analysis, supervision, funding acquisition, visualization, project administration, resources, writing – original draft, writing – review and editing. **Sumei Zhang:** investigation, writing – review and editing. **Xuanbing Tang:** investigation.

## Funding

This work was supported by the Natural Science Basic Research Program of Shaanxi Province (funding recipient: ZL, grant number: 2021JM‐ 498).

## Disclosure

The authors have checked to make sure that our submission conforms as applicable to the Journal's statistical guidelines described here. On the author team, there is an author who has taken a course in nursing statistics and Lu Zhang is the statistics teacher. The authors affirm that the methods used in the data analyses are suitably applied to their data within their study design and context, and the statistical findings have been implemented and interpreted correctly. The authors agree to take responsibility for ensuring that the choice of statistical approach is appropriate and is conducted and interpreted correctly as a condition to submit to the Journal. No artificial intelligence tools were used in the writing, analysis, or interpretation of this study. AI was used for language polishing and editing.

## Conflicts of Interest

The authors declare no conflicts of interest.

## Data Availability

The datasets generated and/or analysed during the current study are available from the corresponding author upon reasonable request.
